# Interlimb Dynamic after Unilateral Focal Lesion of the Cervical Dorsal Corticospinal Tract with Endothelin-1

**DOI:** 10.3389/fnana.2017.00089

**Published:** 2017-10-13

**Authors:** Walther A. Carvalho, Carlomagno P. Bahia, Jéssica C. Teixeira, Walace Gomes-Leal, Antonio Pereira

**Affiliations:** ^1^Pará State University Center, Belém, Brazil; ^2^Laboratory of Neuroplasticity, Institute of Health Sciences, Federal University of Pará (UFPA), Belém, Brazil; ^3^Institute of Biological Sciences, Federal University of Pará (UFPA), Belém, Brazil; ^4^Institute of Technology, Federal University of Pará (UFPA), Belém, Brazil

**Keywords:** hemispheric specialization, handedness, spinal neuromotor modules, corticospinal tract, focal lesion

## Abstract

Handedness is one of the most recognized lateralized behavior in humans. Usually, it is associated with manual superiority regarding performance proficiency. For instance, more than 90% of the human population is considered more skilled with the right hand, which is controlled by the left hemisphere, than with the left. However, during the performance of bimanual tasks, the two hands usually assume asymmetric roles, with one hand acting on objects while the other provides support, stabilizing the object. Traditionally, the role of the two hands is viewed as fixed. However, several studies support an alternate view with flexible assignments for the two hands depending on the task. The supporting role of the hand depends on a closed loop pathway based on proprioceptive inputs from the periphery. The circuit’s efferent arm courses through the dorsal corticospinal tract (dCST) in rodents and terminate on spinal cord interneurons which modulate the excitability of motoneurons in the ventral horn. In the present work, we developed an experimental model of unilateral lesion targeting the cervical dCST with microinjections of the vasoconstrictor endothelin-1 (ET-1) to evaluate the degree of flexibility of forelimb assignment during a food manipulation task. Our results show that just 3 days after unilateral corticospinal tract (CST) injury in the cervical region, rats display severe motor impairment of the ipsilateral forepaw together with a remarkable reversal of motor assignment between the forelimbs.

## Introduction

Given its widespread occurrence across vertebrates, lateralization of CNS structure and function is thought to be associated with a positive selective advantage (Rogers and Andrew, 2002; Rogers et al., 2004; Duboc et al., 2015). One hypothesis is that hemispheric lateralization allows fast, efficient information processing in the brain, improving overall cognition and motor performance (Levy, 1969; Ringo et al., 1994; Knecht et al., 2000; Marzke and Marzke, 2000). Lateralization may also increase brain efficiency by reducing interference between potentially competing processes, such as limb coordination during bimanual tasks (Rogers et al., 2004).

Humans and other mammals can perform complex coordinated forelimb movements during goal-directed actions, such as food manipulation, with action components being unequally distributed across the two arms (Lemon, 2008). The traditional view of motor control holds that the left-hemisphere is specialized for skilled hand movements and thus the performance of the right arm is superior to the left arm in right-handers (Ocklenburg et al., 2014). An alternative theory, however, suggests that each arm/hemisphere is in fact specialized for controlling different movement features during bimanual interaction (Haaland and Harrington, 1989; Sainburg, 2002). Accordingly, in many bimanual tasks, one arm (“nondominant”) is preferably used for stabilization (e.g., holding a fork) while the other arm (“dominant”) performs movements with higher demands for precision (e.g., cutting with a knife). In other words, the nondominant arm excels in impedance control, characterized by corrective, feedback elements, while the dominant arm excels in tasks requiring precise coordination of limb dynamics, which rely on predictive, feedforward elements (Wang and Sainburg, 2007).

Though the distinction between the two control processes, predictive and corrective, is well established in bimanual tasks, the profile observed for the dominant and nondominant arms reflect a default mode that is based on habitual functional requirements rather than an absolute limit in capacity to access the controller specialized for the opposite limb (Johansson et al., 2006; Reuter et al., 2016). This view is supported by the fact that there is also evidence for the lack of manual asymmetries in motor planning of bimanual tasks (for review, see Seegelke et al., 2014).

The voluntary control of skilled arm movements is mediated by the corticospinal tract (CST), which originates from a wide variety of cortical areas, including motor and somatosensory regions (Heffner and Masterton, 1983). In primates, the main CST runs through the lateral columns and contains axons from both cortical hemispheres, while in rodents it is primarily located in the dorsal column and originate from the contralateral cortex (Lemon, 2008). In humans, the CST makes direct contact with cervical motoneurons which innervate forelimb muscles, while in rodents this connection is indirect, via interneurons.

Transection of the dorsal CST (dCST) at cervical segments C1/C2 did not affect skilled reaching and grasping in rats, according to Alstermark and Pettersson (2014). The explanation for this outcome is that in rodents skilled reaching movements are mediated by other descending pathways which are spared by the dCST lesion, namely the lateral CST and the rubrospinal tract (RST; Morris et al., 2011; Morris and Whishaw, 2016). Lesion of the dCST, on the other hand, abolishes the operant conditioning of the H-reflex, the electrical analog of the spinal stretch reflex (SSR; Chen et al., 2002). Through the dCST, the sensorimotor cortex controls long-latency components of the stretch reflex which are relevant to the regulation of limb impedance and postural support during skilled bimanual tasks (Cheney and Fetz, 1984; Pruszynski et al., 2011).

One of the difficulties in understanding the role of specific pathways in controlling skilled forelimb movements is associated with the anatomical organization of the descending tracts. It’s hard to isolate specific pathways and the available experimental lesion models based on transection are not selective and usually cause collateral damage to nearby tissue. They also make it difficult to differentiate the regenerative capacities of different motor pathways after lesions (Ghasemlou et al., 2005).

Endothelin-1 (ET-1) is a peptide produced mostly by endothelial cells (Yanagisawa et al., 1988; Davenport et al., 2016) which cause potent and long-lasting vasoconstriction through the sharp increase in intracellular levels of calcium ions in vascular smooth muscle cells. ET-1 acts on two types of receptors, ETA and ETB, which belong to the family of G protein-coupled receptors and is frequently used to model lacunar stroke, causing circumscribed and reproducible lesions in the brain (Sommer, 2017) and the spinal cord (Benton et al., 2005).

In the present work, we propose a model to selectively lesion the dCST with unilateral microinjections of ET-1. This model can be used to test the prediction that handedness is dynamic and depends on task requirements. As a bimanual task, we used an adaptation of the Vermicelli Handling Test (Pasta Test), which measures dexterous forepaw function in rats (Allred et al., 2008). During the test, rats eat a piece of pasta using an asymmetrical holding pattern, where one paw (dominant) grasps the piece and the other (nondominant) is used for guiding it to the mouth (Allred et al., 2008).

## Materials and Methods

### Subjects

A total of 12 adult Wistar male rats were used in this study. All animals were housed in standard cages on a reverse day-night cycle (12 h—lights off at 8 a.m.) with the temperature set at 25°C and access *ad libitum* to standard rodent chow and water. Animals were acclimated to the housing facility for 1 week before training, and behavioral tests were performed during the dark cycle. The experimental procedures were approved by the Federal University of Pará’s Institutional Animal Care and Use Committee (CEUA/UFPA).

### Experimental Setup

**Figure 1:**
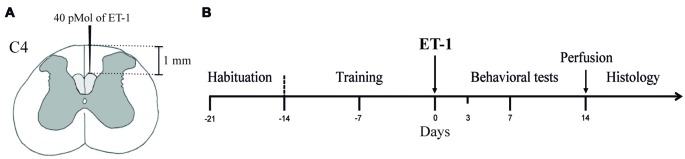
Experimental design. **(A)** Two-hundred and fifty nanoliters of Endothelin-1 (ET-1; 40 pMol) was injected into the dCST at the C4 level. **(B)** Animals of all experimental groups (Sham and Lesion) were submitted to a 1-week acclimatization to the experimental setup and a 2-week training period before surgery for the injection of either sterile saline (Sham Group) or ET-1 (Lesion Group) into the dCSTs. The behavioral tests were performed before (baseline), and 3, 7 and 14 days after surgery. After the behavioral tests, animals were sacrificed and transcardially perfused for histological procedures.

The animals were randomly separated into two experimental groups which were given microinjections of either sterile saline (Sham Group, *n* = 6) or ET-1 (Lesion Group, *n* = 6) in the dCST (Figure 1A). The animals were habituated to the test apparatuses for 1 week once a day for at least 15 min to avoid neophobic behavior (Figure 1B). In the following 2 weeks, the animals were trained to perform the Pasta Test (Allred et al., 2008) and the Montoya Staircase Test (Montoya et al., 1991). During the training sessions, the preferential use of either the left or right forepaw was recorded (Whishaw and Coles, 1996; Ballermann et al., 2001).

### Surgical Procedures

The animals were anesthetized with a mixture of ketamine hydrochloride (VETANARCOL^®^, KÖNIG, 72 mg/kg) and xylazine hydrochloride (KENSOL^®^, KÖNIG, 9 mg/kg; IM). A single dose of 0.1 mg/kg of atropine sulfate (PASMODEX^®^, ISOFARMA, 0.25 mg/ml; IM) was administered to decrease mucus production in the airways during anesthesia. Body temperature was maintained at 37°C with the use of a heating pad. The animal was positioned in a stereotaxic apparatus for ET-1 injection.

After a posterior cervical vertebral laminectomy, we injected 40 pMol of ET-1 (SIGMA-ALDRICH) diluted in 250 nL of sterile saline in the dCST using a graduated glass micropipette (SIGMA-ALDRICH, HIRSCHMANN). The microinjection was performed at a depth of 1 mm from the dorsal pial surface, medial to the posterior spinal artery (Figure 1A). After the injection, the micropipette remained in the spinal cord for 5 min to prevent reflux.

### Behavioral Tests

The tests were carried out before the surgery (baseline), and 3, 7 and 14 days after the ET-1 injection. We evaluated interlimb asymmetries of skilled forepaw and finger movements with the Pasta Test (Allred et al., 2008) and independent forelimb reaching and grasping with the Montoya Staircase Test (Montoya et al., 1991). In the Pasta Test, rats handle a piece of spaghetti asymmetrically by grasping the 70 mm segments with one paw, while using the other paw to guide the spaghetti towards the mouth (Allred et al., 2008; Tennant et al., 2010). In this work, the paw the animal used to grasp the pasta in at least 80% of occurrences during the habituation phase was called preferred paw (PP), and the other was called non-PP (NPP; Whishaw and Coles, 1996; Ballermann et al., 2001).

The PP makes the following movements during the test: Extension/Flexion (Ext/Flex) and Abduction/Abduction (Abd/Add) movements. Meanwhile, the NPP makes only Release/Contact (Rel/Recon) movements. In the Staircase Test, two pellets of sugar were deposited in each step, totaling 14 pellets on each side of the staircase (left and right). The rats were placed on the top of the platform for 15 min and the number of pellets recovered during this time was used to calculate the animal’s percentage of success (number of pellets recovered/total number of pellets).

### Histology

**Figure 2:**
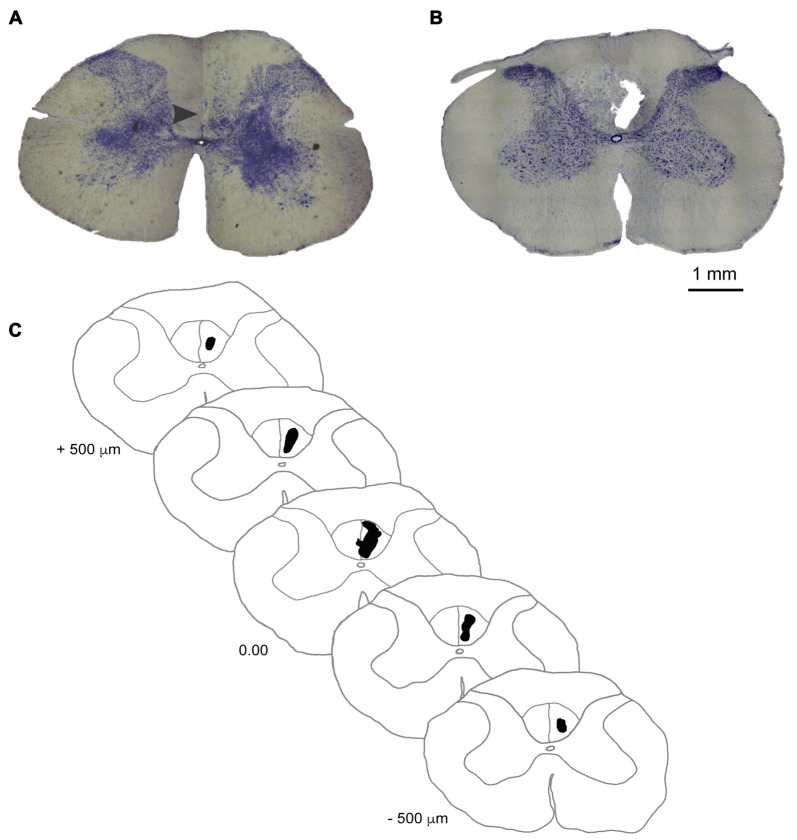
Ischemic damage to the dCST. **(A)** Photomicrograph of a coronal section of the dCST stained with Nissl showing the location (arrowhead) of a vehicle injection in an animal of the sham group 14 days after the procedure. **(B)** Photomicrograph of a coronal section of the dCST stained with Nissl showing the epicenter of the ET-1 lesion marked by the presence of a fluid-filled cavitaty in the dCST 14 days after the procedure. **(C)** Diagram of a representative ET-1 injury to the dCST in a reconstructed series of Nissl-stained sections. Scale bar: 1 mm.

After behavioral tests, the animals (*n* = 6/group) were deeply anesthetized by an intraperitoneal administration of a lethal dose of Ketamine (80 mg/Kg) and transcardially perfused with heparinized phosphate-buffered saline (PBS) followed by 4% paraformaldehyde (PFA) in PBS. Blocks of tissue containing the lesion site in the spinal cord’s cervical (C2–C6) segments were dissected, post-fixed in 4% PFA (24 h at 4°C), and cryoprotected through immersion in 0.1 M phosphate buffer with increasing sucrose concentrations and then frozen in Tissue-Tek O.C.T. (Sakura Finetek, Torrance, CA, USA). The block was sectioned coronally into 50 μm-thick sections with a cryostat microtome (Microm HM 520, Thermo Fisher Scientific, Waltham, MA, USA) and sections were collected to determine infarct volume. Every section at 250 μm intervals was Nissl-stained (Figure 2) to measure the infarct volume in half of the animals of the lesion group (*n* = 3). Infarct volume was estimated with ImageJ (NIH, downloaded from http://imagej.nih.gov/ij/) and calculated according to a methodology described by Nguemeni et al. (2015).

### Statistical Analysis

The statistical analysis was performed using analysis of variance (ANOVA) followed by the Tukey’s test. The significance level was 0.05. The researchers conducting the analyses were blind to the experimental groups.

## Results

### Infarct Volume

Animals from the sham group did not have significant morphological alterations in the spinal cord when evaluated on the 14th day after surgery (Figure 2A). After the same period, the ET-1 injection site in animals from the lesion group is characterized by an ischemic core with maximal tissue damage and complete loss of identifiable cellular structures (Figure 2B), as seen in other studies in different regions of the brain (e.g., Cooperrider et al., 2014). This degree of tissue loss is distinct from a study where ET-1 was injected into the spinal cord’s gray matter (Benton et al., 2005). One of the reasons may be the smaller concentration of ET-1 used by those authors. However, we cannot also rule out the possibility that some tissue loss ensued from the action of mechanical forces during histological procedures. The average infarct volume in the lesion group (*n* = 3) was 0.016 ± 0.0013 mm^3^. A serial reconstruction of coronal sections of a representative animal shows the extension of the lesion along the longitudinal axis of the cervical spinal cord (Figure 2C).

### Behavioral Tests

**Figure 3:**
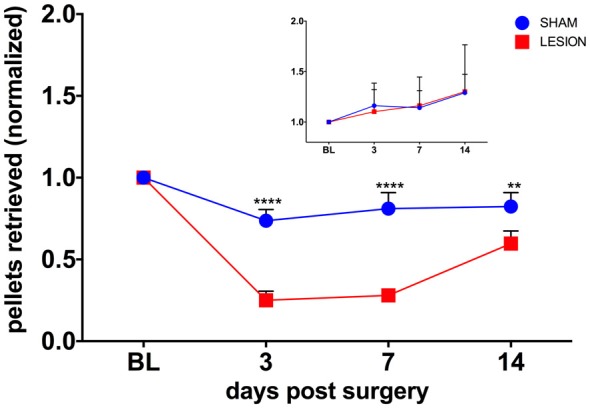
The dCST lesion impaired the performance of the preferred paw (PP), but not the non-preferred paw (NPP), in the Montoya staircase test. The performance of the PP of animals from the lesion group was lower than the sham group in all time points. However, the performance of the NPP was similar between the groups (inset). Values were normalized to baseline (BL) levels. ****p < 0.0001, **p = 0.0024. Two-way analysis of variance (ANOVA), repeated measures, Sidak’s multiple comparisons test. Error bars represent mean ± SEM.

To test whether the unilateral lesion of the cervical dCST with ET-1 impairs forelimb function, we compared the effects of injecting either ET-1 or vehicle on the unimanual performance in the Montoya staircase test. We found the baseline performance of the test with the PP is significantly higher than with the NPP for both the sham (PP = 9.33 ± 0.33, NPP = 6.00 ± 0.52, *t*_(10)_ = 5.42, *p* = 0.0003) and the lesion (PP = 9.67 ± 0.56, NPP = 5.17 ± 0.48, *t*_(10)_ = 6.130, *p* = 0.0001) groups. A two-way repeated measures ANOVA was conducted to evaluate the effect of group (sham, lesion) and days after surgery (3, 7, 14) on the number of pellets retrieved in the Montoya test with the PP (Figure 3). There was a statistically significant interaction between the effects of the factors group and days after surgery on the number of pellets retrieved, *F*_(3,15)_ = 19.96, *p* < 0.0001 (Figure 3). The performance of the PP was significantly lower for the lesion group, when compared to the sham group, at all time points (*p* = 0.0024; Figure 3). The performance of the NPP, however remained similar between groups at all time points (*p* = 0.971).

**Figure 4:**
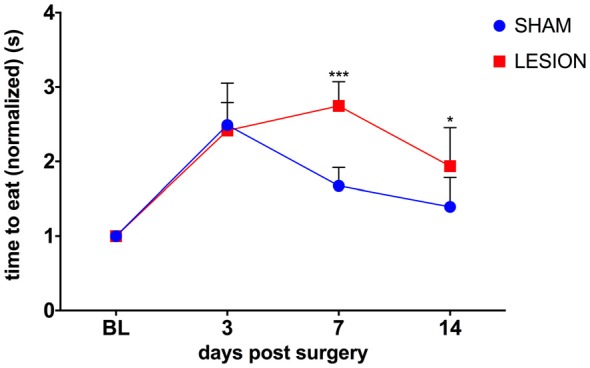
dCST lesion caused a transient decrease in the performance of the pasta test. Animals from the sham group recovered faster from the surgery than dCST-lesioned animals. The unilateral lesion of the dCST with ET-1 causes a more persistent deficit. ***p < 0.001, *p < 0.05. Two-way ANOVA, repeated measures, Tukey’s multiple comparisons test. Error bars represent mean ± SEM.

To test whether the unilateral lesion of the cervical dCST with ET-1 impairs skilled bimanual forelimb movements, we compared the effect of the injection of either ET-1 or vehicle into the dCST on bimanual performance in the pasta test. A two-way repeated measures ANOVA was conducted to evaluate the effect of group (sham, lesion) and days after surgery (3, 7, 14) on the average time taken to consume a piece of pasta (Figure 4). There was a statistically significant interaction between the effects of the factors group and days after surgery on the time to eat the piece of pasta, *F*_(3,15)_ = 8.1, *p* < 0.0019 (Figure 4). The performance of both sham (*p* < 0.0001) and lesioned animals (*p* = 0.0101) decreased 3 days after the surgery. However, at day 7, sham animals began to recover (*p* = 0.0101), while lesioned animals maintained the same level of impairment (*p* < 0.0001), compared to baseline. Only at day 14 lesioned animals showed some recovery (*p* = 0.001), while the sham group returned to baseline levels (*p* > 0.05; Figure 4).

**Figure 5:**
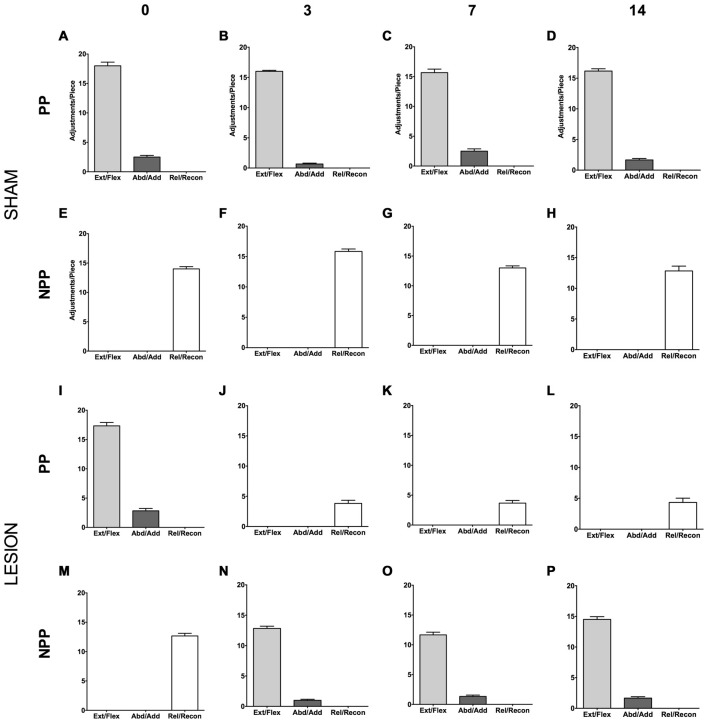
Effect of dCST lesion on the pasta test. The histograms depict the average number of motor adjustments performed with the PP and NPP, both in the Sham **(A–H)** and the Lesion Group **(I–P)**. Error bars represent mean ± SEM.

Animals from the Sham group displayed an asymmetric motor performance in the pasta test (see Figures 5A–H), similar to other studies (Allred et al., 2008; García-Alías et al., 2009). The same was true for the lesion group before surgery (see Figures 5I,M). Three days after surgery, however, the animals presented compensatory adjustments, with the NPP assuming the movements lost in the PP after the focal ischemic lesion in the dCST (see Figures 5J,N). We observed the same pattern after 7 (Figures 5K,O) and 14 days post lesion (Figures 5L,P).

**Figure 6:**
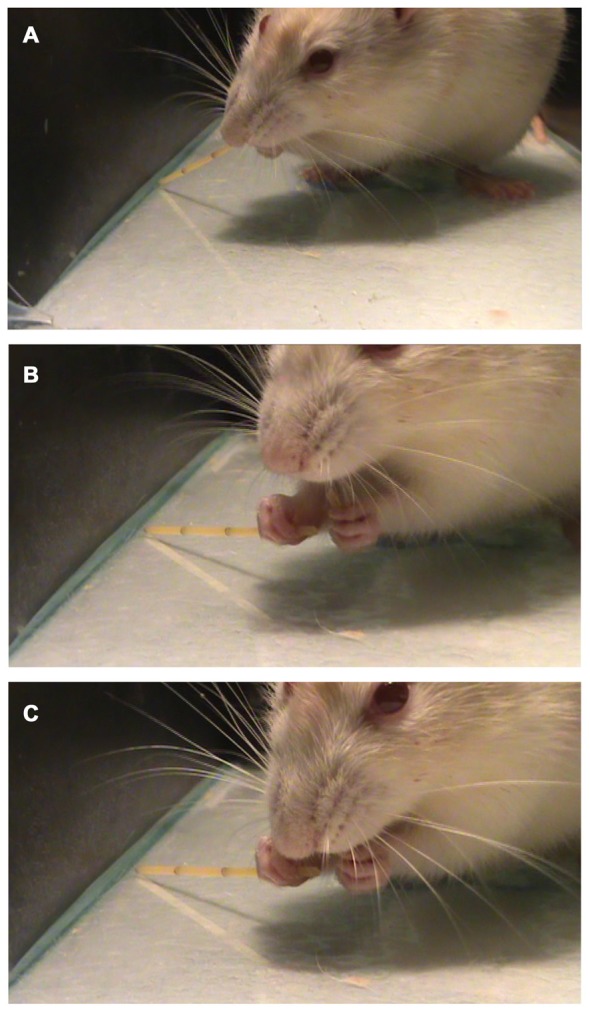
Performing the pasta Test. A representative trial of the pasta test showing a previously unreported behavior called “*Break and eat while grasping*” in which the animal grasps **(A)** and breaks the piece of pasta with the mouth **(B)** and then eats it **(C)**, holding the two pieces of pasta at the same time.

Allred et al. (2008) described a list of ten atypical movements that can be observed in the pasta handling test. In the present work, we found only two of those movements (*Drop* and *Failure to Contact*). Also, we describe a new atypical movement called “*break and eat while grasping*” (Figures 6A–C) when the animal breaks a piece of pasta and eat it while holding the other piece.

## Discussion

Functional lateralization is a basic organizing principle of the mammalian brain (Vallortigara and Rogers, 2005). For instance, humans and other mammals display a lateralized preference in forelimb use during the performance of skilled bimanual movements. Traditionally, one of the hands is considered to be more skilled than the other, and this is reflected in the concept of hand dominance, with a majority of people (>80% of the population) preferring to use the right hand over the left (Gilbert and Wysocki, 1992; Annett, 1996). An alternative view suggests that hands are in fact specialized for different roles in bimanual movements and controlled by either feedforward or feedback commands to initiate prepared actions or to provide stabilization, respectively.

In the present work, we performed a unilateral lesion of the dCST with ET-1 in rats and evaluated the effect on bimanual movements associated with a food handling task, the pasta/vermicelli test (Allred et al., 2008), for 2 weeks. To increase the precision of our lesions, we used a concentration of ET-1 which is 20× lower than the lowest dose employed before (Windle et al., 2006; Nguemeni et al., 2015). Other lesion studies available in the literature (e.g., Whishaw et al., 1992; Alaverdashvili and Whishaw, 2008) resorted to transection techniques and were not able to selectively target the dCST, either leaving other descending projections intact or producing incomplete lesions.

As shown in Figure 3, the unilateral lesion of the cervical dSCT had just a transient effect on the execution of reaching movements with the ipsilateral forelimb during the staircase task. The performance of reaching movements returned to baseline levels at the end of the observation period of 2 weeks. Just 3 days after the lesion, animals already used the affected forepaw, though with an average rate of success measured in pellet recovery of less than 40% of baseline levels and also 50% lower than the performance of sham animals. The rate of success steadily increased towards baseline levels until the 14th post-lesional day. Monkeys also had a similar recovery profile in reaching movements after unilateral lesion of the CST, which in primates runs down the lateral funiculus in the spinal cord, together with the RST (Sasaki et al., 2004).

The dCST in rats is associated with the operant conditioning of the H-reflex, the electrical analog of the SSR (Chen et al., 2002). Learning of skilled movements is linked with long-term changes in the amplitude of the H-reflex (Wolpaw and Lee, 1989). The persistent decrease in the H-reflex amplitude associated with learning is probably due to the strengthening of the inhibitory influence of the dCST on ventral motoneurons via inhibitory interneurons (Chen and Wolpaw, 2002). Changes in the reflex excitability parallel skill acquisition and more precise control of movement performance, with smaller reflexes being associated with more accurate movements (Nielsen et al., 1993). After the dCST of rats is lesioned bi-laterally at vertebral level T8-T9, the H-reflex amplitude increased only briefly for the first 1–2 days immediately after transection, but returned to basal levels afterward (Chen et al., 2001). After unilateral pyramidal tract session, there is a sprout of proprioceptive afferents into cervical gray matter regions deprived of dCST terminations, together with a reduction in the frequency-dependent depression of the H-reflex, suggesting hyperreflexia or spasticity (Tan et al., 2012).

Despite the interlimb asymmetries in the execution phase of bimanual movements, the two hands seem to have equal performance capabilities. Motor planning, for instance, is performed in an effector-independent manner (Wong et al., 2015). Effector attribution for execution of bimanual movements depends on task constraints (Bryden et al., 2007) and lifelong practice (Seegelke et al., 2014). This view is supported by our results showing that the unilateral lesion of the dCST causes a switch of the corrective and predictive roles of forelimbs in a bimanual task. Both the sham and the lesion group are significantly slower to handle pieces of pasta immediately after surgery (see Figure 4). However, animals in the lesion group continue to present deficits in the task at least until the last experimental observation, 2 weeks after surgery. This difference in behavior suggests that the initial difficulty in performing the task presented by animals in the sham group is associated with the surgical procedures. The lesion group, however, shows persistent deficits in the task, at least 9 days longer than the sham group. Just 3 days after the lesion, the affected dominant arm assumes the stabilization role played by the nondominant arm, while the latter displays the same complement of goal-directed movements of the former. This new arrangement persists for the whole observation period of 2 weeks after the lesion. After the unilateral dCST lesion, however, there was a significant, long-lasting increase in the time animals spent to consume each piece of pasta (see Figure 4).

## Conclusion

We conclude that unilateral focal lesions to the cervical dCST do not have long-term effects on the performance of reaching movements of the forelimb in rats. However, the loss of corticospinal inputs to spinal cord circuits lead to adjustments in the role played by each forelimb during goal-directed bimanual movements. This effect is probably related to hyperreflexia and spasticity in the affected member due to the loss of dCST inhibition of dorsal horn neurons and the subsequent sprouting of primary afferent fibers to this region (Tan et al., 2012). Our results are important to understand the genesis of motor asymmetries in bimanual movements and to test the effects of potential neuroplasticity-inducing therapies, which aim at the recovery of arm movements affected by injury to the spinal cord.

## Author Contributions

WAC, CPB and AP designed the study and wrote the manuscript; WAC and JCT collected the data; WAC, CPB, WG-L and AP analyzed the data; all authors read and commented on the manuscript.

## Conflict of Interest Statement

The authors declare that the research was conducted in the absence of any commercial or financial relationships that could be construed as a potential conflict of interest.
